# A systematic review of 47 published cases of extramedullary hematopoiesis in myelofibrosis: clinical patterns, treatment responses, and prognostic implications

**DOI:** 10.1007/s00277-026-07146-2

**Published:** 2026-06-30

**Authors:** Andrea Duminuco, Veronica Vecchio, Giulio Lavenia, Patrick Harrington, Annalisa Santisi, Arianna Sbriglione, Giuseppe A. Palumbo

**Affiliations:** 1 Dipartimento di Scienze Mediche Chirurgiche e Tecnologie Avanzate “G.F. Ingrassia”, University of Catania, Catania, Italy; 2 Hematology with BMT Unit, A.O.U. “G. Rodolico-San Marco”, Catania, Italy; 3 Department of Haematology, Guy’s and St Thomas’ NHS Foundation Trust, London, UK

**Keywords:** Myelofibrosis, Extramedullary hemopoiesis, JAKi, Real-life, Case series

## Abstract

Extramedullary hematopoiesis (EMH) in myelofibrosis manifests beyond classical splenomegaly, causing life-threatening complications, yet lacks systematic characterization in the JAK inhibitor era. We systematically reviewed 36 publications (2011–2025), available on PubMed database, reporting 47 unique myelofibrosis-associated EMH cases, analyzing demographics, anatomic distribution, presentations, diagnostics, treatments, and outcomes.

Median age was 68 years (range 43–82; M: F 26:21); primary myelofibrosis predominated (64%). EMH favored atypical sites: abdominopelvic (28%), thoracic (26%; pulmonary/pleural), serosal (17%), cutaneous (13%). Symptomatic onset occurred in 75% (dyspnea 28%, abdominal pain 21%). CT/MRI revealed characteristic lobulated masses with T2 hypointensity; biopsy confirmed trilineage hematopoiesis with atypical megakaryocytes (CD61+). Ruxolitinib-based regimens, administered in 19 patients (40%) as EMH-directed therapy, yielded rapid palliation (dyspnea/ascites resolution), complemented by radiotherapy (11%) and splenectomy; HSCT achieved complete remission in one case. Mortality reached 38% (median follow-up 5 months), driven by EMH-related organ failure or AML transformation. Atypical EMH signals advanced myelofibrosis with dismal prognosis, responsive to JAK inhibitors/locoregional therapies but highlighting sanctuary biology. Systematic surveillance and transplant referral are imperative; prospective registries are needed to refine risk models and test novel antifibrotics.

**Supplementary Information:**

The online version contains supplementary material available at https://doi.org/10.1007/s00277-026-07146-2.

## Introduction

Primary myelofibrosis (PMF) is a Philadelphia chromosome-negative chronic myeloproliferative neoplasm (MPN) defined by clonal proliferation of atypical megakaryocyte precursors, constitutive JAK-STAT pathway activation, progressive bone marrow fibrosis, and extramedullary hematopoiesis (EMH) [1, 2]. Current therapeutic management of MF relies primarily on JAK inhibitors, which effectively palliate splenomegaly and constitutional symptoms in most patients but exhibit limited cytoreductive activity and frequently worsen anemia [3–6]. Allogeneic hematopoietic stem cell transplantation offers the only curative potential for eligible candidates, though extramedullary hematopoiesis often complicates disease trajectory and response assessment [7–9].

EMH emerges as a compensatory mechanism when the reticulin/collagen-saturated marrow microenvironment fails to support hematopoiesis: clonal hematopoietic stem/progenitor cells, guided by chemokine gradients (e.g., CXCL12), traffic to permissive ectopic niches, recapitulating trilineage foci (erythroid, myeloid, megakaryocytic) [10, 11].

**Fig. 1:**
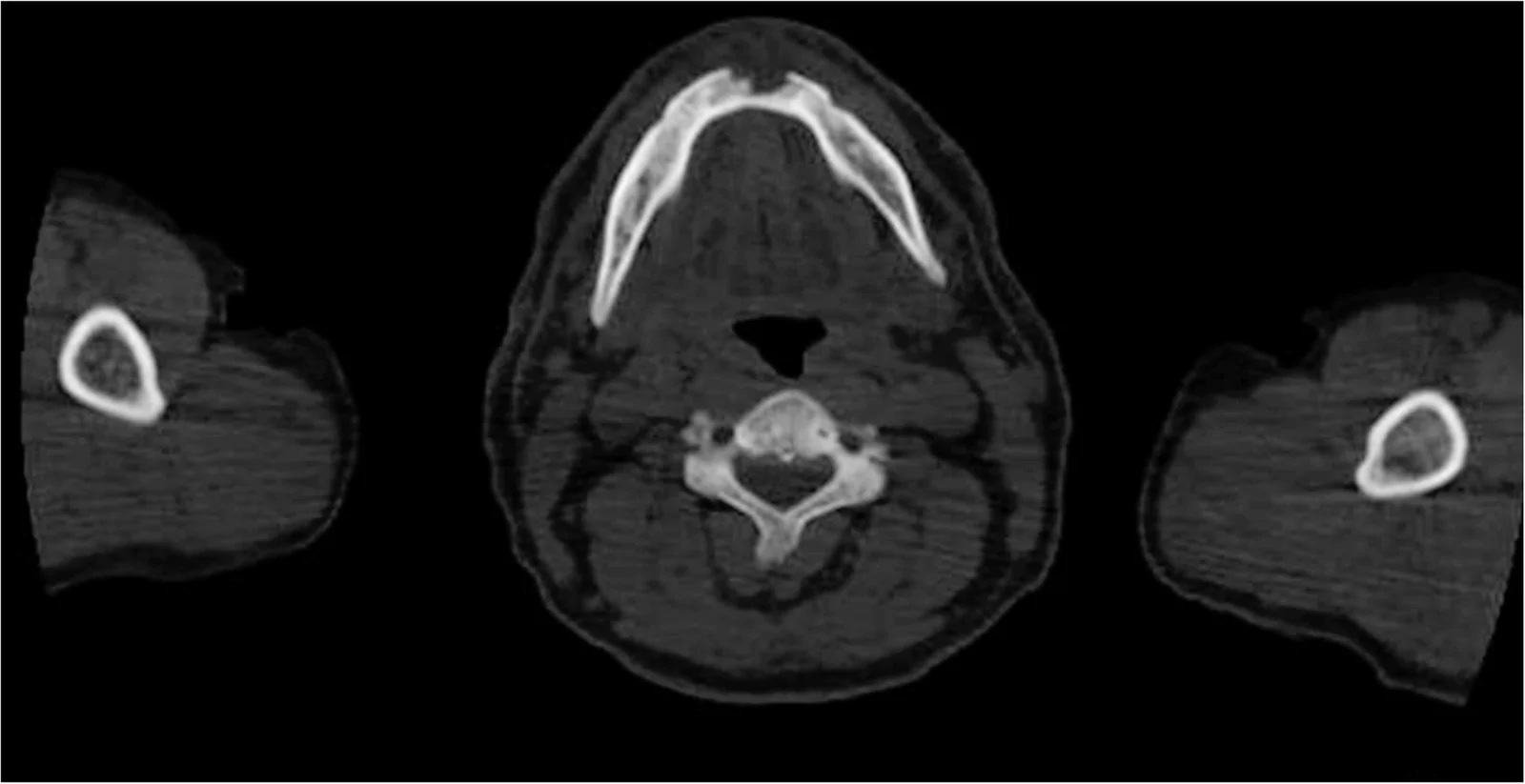
Localization of extramedullary hematopoiesis, as described in the text

We report a 60-year-old man with JAK2 V617F-mutated PMF (grade 3 fibrosis, favorable del(20q)) who was initially managed conservatively after diagnosis in July 2011. By May 2020, progressive symptomatic splenomegaly (16 cm below the costal margin) and a Total Symptom Score of 23 prompted initiation of ruxolitinib 20 mg twice daily, resulting in an initial reduction in spleen volume and improved performance status. In 2023, contrast-enhanced CT detected a heterogeneous mandibular soft-tissue mass (30 × 38 mm) with bone lysis and muscular extension, which was confirmed as extramedullary hematopoiesis by biopsy (Fig. 1). Sequential focal radiotherapy achieved only partial control.

Next-generation sequencing revealed high-risk mutations (JAK2 exon 14, SRSF2 exon 1, KRAS exon 3), correlating with ruxolitinib resistance, transfusion dependence (4 red cell units/month), worsening anemia (Hb 8.0 g/dL), asthenia, and spleen regrowth (8 cm below the costal margin) by May 2024. Ruxolitinib was discontinued, and compassionate momelotinib 200 mg daily was started in June 2024. Within 3 weeks, hemoglobin rose to 10.4 g/dL, transfusion requirement resolved, and systemic symptoms (asthenia, night sweats, weight loss) improved markedly. After 3 months, PET demonstrated a metabolic response (SUVmax reduced from 14 to 4 at the mandibular site), ultrasound confirmed spleen shrinkage to 13 cm, and tolerance was excellent (transient mild headache). Conditioning for allogeneic hematopoietic stem cell transplantation from an HLA-identical sibling donor was successfully initiated with Hb 10.2 g/dL and good performance status, positioning momelotinib as an effective bridge therapy for refractory EMH and multisystem disease progression.

EMH complicates up to 50% of MF cases at autopsy, manifesting predominantly as splenomegaly/hepatomegaly but with atypical sites (pulmonary, renal, neurologic, serosal) increasingly reported, especially in advanced disease or post-JAK inhibitor therapy [12]. A recent literature review highlights EMH’s diverse sequelae, from indolent incidentalomas to life-threatening organ failure via mass effect, vascular occlusion, or cytokine storm, often discordant with spleen/symptom response, suggesting clonal escape or sanctuary biology [13]. Prognostication remains challenging, with atypical EMH signaling associated with accelerated progression and complicating transplant eligibility.

Systemic therapies target the underlying MF with JAK inhibitors (ruxolitinib, fedratinib, pacritinib, momelotinib), achieving spleen/liver EMH reduction and symptom palliation (30–50% reduction in spleen volume), although atypical sites often persist. Cytoreductive agents (hydroxyurea) provide only modest control. Locoregional interventions, such as low-dose radiotherapy (effective for focal masses), splenectomy, or resection, provide rapid relief, with JAKi and radiotherapy combinations enhancing durability [14].

However, despite anecdotal successes with RT, splenectomy, or novel agents, standardized data on patterns, predictors, and outcomes are lacking, fueling publication bias toward dramatic cases.

We aimed to aggregate individual patient data from PubMed publications to delineate the clinical spectrum, diagnostic features, treatment responses, and survival outcomes of MF-associated EMH, thereby informing risk stratification and therapeutic decision-making.

## Materials and methods

### Study design and ethical considerations

A retrospective systematic review of published case reports and small case series describing EMH in patients with myelofibrosis was conducted. The study was designed in accordance with general principles for systematic reviews of case reports and complied with the Declaration of Helsinki. No ethics approval was required because only anonymized, published data were analyzed.

### Literature search strategy

Literature search was performed through a structured electronic query of the PubMed database, targeting all relevant reports published within the last 15 years (from January 1, 2011 to December 31, 2025), and no prospective hand-searching beyond this date was performed. The primary search strategy combined terms related to EMH and myelofibrosis using Boolean operators: (“extramedullary hematopoiesis” OR “extramedullary hemopoiesis”) AND (“myelofibrosis” OR “myelofibrosis primary” OR “post-PV myelofibrosis” OR “post-ET myelofibrosis”). No language restrictions were initially applied, but only articles available in English with sufficient extractable data were ultimately included. This review was conducted in accordance with the PRISMA 2020 guidelines, and the flow diagram is provided in Supplementary Fig. 1.

### Study selection

Eligible studies included case reports and case series meeting all of the following criteria: patients with a confirmed diagnosis of primary MF or post-polycythemia vera/post-essential thrombocythemia MF according to contemporaneous diagnostic criteria; documentation of EMH in at least one anatomical site outside the bone marrow, confirmed histologically/cytologically or deemed highly likely based on imaging and clinical context; and sufficient clinical detail to extract basic demographic data, EMH site(s), management, and outcome. Cases without a clear myelofibrosis diagnosis (such as EMH in other myeloproliferative neoplasms without fibrosis or non-MPN conditions), reviews, editorials, conference abstracts lacking individual-level data, and experimental or animal studies were excluded. Title, abstract screening, and full-text assessment were performed independently by two reviewers. Discrepancies were resolved by discussion and, where necessary, by adjudication from a third reviewer. Inter-reviewer agreement was not formally quant, ified given the small number of eligible records. Cases reported as “myelofibrosis” without explicit subtype specification were retained, provided the published report documented a confirmed MF diagnosis according to contemporaneous WHO or ICC criteria. These cases were coded as “not specified” (NS) and were not excluded, as the absence of subtype detail in case reports reflects publication practice rather than diagnostic uncertainty.

### Data extraction

Data extraction was performed using a standardized electronic form capturing patient demographics (age at EMH diagnosis, sex); underlying disease characteristics (MPN subtype, time from MF diagnosis to EMH, risk category, cytogenetics); molecular profile (driver mutations); EMH details (anatomical site); clinical presentation (symptoms, incidental vs. symptomatic, life-threatening complications); diagnostic work-up (imaging modalities, histologic/cytologic confirmation, immunohistochemistry/molecular studies); treatments (MF-directed therapy at EMH onset, EMH-specific interventions); and outcomes (EMH response, disease progression, survival status, time to last follow-up or death, cause of death). Ambiguous data were coded as “not reported” to avoid assumptions. The case described in the introduction had been previously published by members of the authorship and included in the analysis, and it was identified through the same PubMed search and extracted using the identical standardized form, with no additional unpublished data incorporated.

### Data synthesis and statistical analysis

Data synthesis focused on descriptive data synthesis. Continuous variables were summarized as medians with ranges, while categorical variables were reported as frequencies and percentages. For cases with reconstructible individual time-to-event data, OS from EMH diagnosis was estimated via Kaplan-Meier methodology, performed as an exploratory descriptive exercise only given that publication date was used as a surrogate for last follow-up in cases where this was not explicitly reported. Prespecified exploratory subgroup analyses examined survival and patterns by EMH site (classical vs. atypical), single- vs. multi-site involvement, JAK inhibitor use at onset, and treatment type (disease-directed vs. supportive). These analyses were interpreted as hypothesis-generating due to heterogeneity and small subgroup sizes. Statistical analysis was performed using MedCalc^®^ Statistical Software version 22.003 (MedCalc Software Ltd, Ostend, Belgium; https://www.medcalc.org; 2023).

## Results

### Overall characteristics

A total of 36 publications reporting 47 individual cases of extramedullary EMH in patients with MF were included in the analysis [15–49]. The majority of reports were single-case reports, with a minority comprising small case series (≥ 2 patients per article; *n* = 5). The median publication year was 2022 (range, 2011–2025), with an apparent increase in reports over the last 4 years.

The median age at EMH diagnosis was 68 years (range, 43–82), and the male-to-female ratio was 26:21. Underlying MPN subtype was PMF in 30 (64%) of cases, post-PV MF in 7 (15%), and post-ET MF in 6 (13%); in 4 (8%) of patients, the precise MPN subtype was not specified. Driver mutation status was available in 28 (59.6%) cases. JAK2 V617F in 19 (40%), CALR in 2 (4.3%), MPL in 1 (2.2%), and triple-negative in 6 (12.7%). Cytogenetic risk and prognostic scores (IPSS for PMF and MYSEC-PM in secondary) were reported only in 29 patients (61.7%), with 1 (2.2%) classified as low-, 12 (25.5%) Intermediate-1, 11 (23.4%) Intermediate-2 and 5 (10.6%) high-risk.

At the time of EMH diagnosis, the median interval from initial MF diagnosis (available for 25 patients) was 4 years (range, 1–15), while 10 (21.3%) patients had a concomitant EMH/MF diagnosis. Disease-modifying or cytoreductive therapy at EMH onset (available for 27 cases) included hydroxyurea in 18 (38.3%), JAK inhibitors (ruxolitinib) in 6 (14%), busulfan/interferon/thalidomide in 1 (2.2%), and no active therapy/transfusion support in 8 (17%). No prior bone marrow transplant was reported.

### Anatomical distribution of EMH sites

Overall, EMH involved a broad spectrum of anatomical sites. Although the most frequent locations are abdominal organs, particularly the liver and spleen, they were reported in only 5 patients. In total, 16 distinct EMH sites were documented.

Abdominopelvic sites emerged as the most prevalent compartment (*n* = 13, 27.6%), predominantly renal and urinary tract involvement (*n* = 5; interstitial kidney tissue, right renal pelvis mass, bilateral hilar/pelvic soft tissue masses, pericalyceal mass with para-aortic lymphadenopathy), followed by gastrointestinal tract EMH (*n* = 4; gastric mucosa/lower body, bile duct, colonic ulcers excluding terminal ileum) and omental/periportal masses (*n* = 2). Inguinal and solitary lymph node enlargement each contributed 1 case.

The thoracic involvement accounted for 25.5% (*n* = 12), encompassing pulmonary lesions (alveolar airspace and pulmonary artery infiltration, parenchymal nodules in right upper/lower lobes; *n* = 5) and pleural deposits (*n* = 4), alongside isolated pericardial EMH (*n* = 1) and mediastinal lymph nodes (*n* = 2).

Cutaneous and soft tissue manifestations were documented in 6 cases (12.8%), including scalp lesions (*n* = 1), multiple violet-colored papular patches on trunk/extremities (*n* = 1), and cutaneous nodules (*n* = 4). Serosal and fluid compartments represented 17% (*n* = 8), with EMH in serous fluids (pleural effusions *n* = 5, ascitic fluid *n* = 1, cerebrospinal fluid *n* = 1) and an isolated ascitic aspirate (*n* = 1).

Endocrine/glandular sites included thyroid EMH (*n* = 4) and ocular involvement (*n* = 1), while central nervous system disease comprised spinal cord infiltration (*n* = 1) and subdural hematoma with EMH membrane (*n* = 1). Paravertebral thoracic masses (Th5–Th10; *n* = 1) and multifocal presentations spanning multiple systems (*n* = 3) completed the spectrum, confirming the propensity for EMH dissemination beyond classical reticuloendothelial organs in MF.

**Fig. 2:**
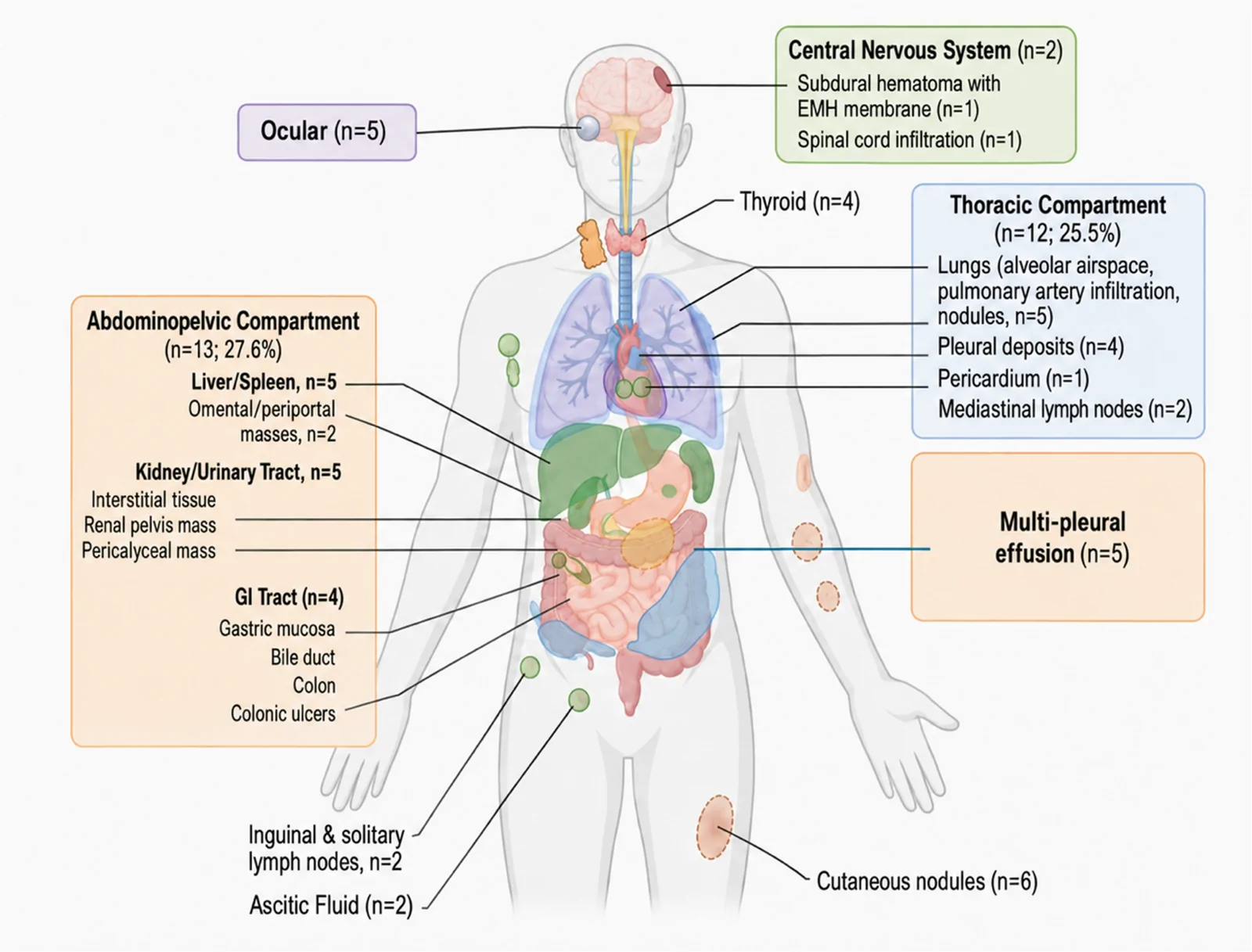
Anatomical spectrum of extramedullary hematopoiesis, as described in the text

Anatomical distribution is reported in Fig. 2.

### Clinical presentation and diagnostic work-up

EMH presentation ranged from incidental findings to life-threatening complications. It was diagnosed incidentally in 12 patients (25.5%), typically during imaging for unrelated complaints or routine surveillance, while symptomatic presentation occurred in 35 cases (74.5%), with manifestations attributable to local mass effect, organ dysfunction, or systemic disease burden.

Respiratory symptoms were predominant (*n* = 13, 27.7%), encompassing dyspnea (*n* = 8), cough (*n* = 4, 8.5%), and hemoptysis (*n* = 2), often linked to pulmonary, pleural, or pericardial EMH. Abdominal complaints affected 10 patients (21.3%), including intermittent right lumbar or generalized abdominal pain (*n* = 4), distension (*n* = 3), nausea/vomiting (*n* = 2), and hematemesis/tarry stools (*n* = 1), typically associated with renal, omental, or gastrointestinal involvement.

Constitutional/B symptoms were reported in 11 cases (23.4%), featuring fatigue (*n* = 6), weight loss (*n* = 5), fever/night sweats (*n* = 4), and generalized pruritus (*n* = 1). Renal/urologic dysfunction emerged in 5 cases (10.6%), manifesting as nephrotic syndrome, hematuria with recurrent urinary tract infections, or deterioration of kidney function with proteinuria. Neurologic symptoms included disturbed consciousness, refractory postoperative bleeding, and spinal compression syndromes at T3–T8 or T10–T12 levels (*n* = 3).

Less common presentations involved ocular lesions with reduced visual acuity (*n* = 1), palpable subcutaneous nodules (*n* = 1), epistaxis (*n* = 1), jaundice/hepatomegaly/portal hypertension (*n* = 2). Lymphadenopathy was found in those with lymph node localization. Rapid weight gain (*n* = 1) and colitis (*n* = 1) were reported as thyroid and gastrointestinal involvement, respectively. Multifocal or overlapping symptoms such as weakness, chills, ecchymosis, and hyporexia were noted in several patients, often reflecting advanced systemic disease rather than isolated EMH effects.

Diagnostic assessment of extramedullary hematopoiesis was primarily imaging-driven through cross-sectional imaging, with computed tomography (CT) utilized as the initial modality in 29 cases (61.7%), magnetic resonance imaging (MRI) in 8 cases (17%), ultrasound (US) in 6 cases (12.8%), chest X-ray in 5 cases (10.6%), and PET/CT or scintigraphy (111In) in 3 cases (6.4%); multiple modalities were combined in 12 cases (25.5%).

CT features were remarkably heterogeneous. Pulmonary EMH appeared as bilateral bronchovascular consolidations, ground-glass opacities, consolidative areas in lingula/right lobes, or right pulmonary artery filling defects extending into middle lobe parenchyma (*n* = 8). Renal/peri-renal lesions presented as mildly enhancing masses in the renal pelvis, parapyelic/perihilar solid nodules, or diffuse kidney enlargement with increased echogenicity on US (*n* = 5). Abdominal CT revealed multi-conglomerated enlarged inguinal/mediastinal/hilar lymph nodes, diffuse mosaic attenuation with vascular enlargement, submucosal gastric edema/hyperemia, esophagogastric varices, hypodense periportal lesions, and chronic left subdural hematoma with midline shift (*n* = 12). Thyroid nodules showed annular peripheral calcification, multinodular goiters with microcalcifications/cervical lymphadenopathy, or isolated nodules without calcification (*n* = 4).

MRI characteristics included marked low signal intensity on both T1- and T2-weighted sequences for parapyelic/perihilar renal lesions (*n* = 2) and periportal hepatic masses (*n* = 1), reflecting the hemosiderin content typical of hematopoietic tissue; additional MRI depictions involved unspecified spinal or paravertebral findings (*n* = 3). Chest X-ray was primarily employed for acute respiratory presentations, demonstrating peri-hilar opacities, lingular opacities, or consolidations with ipsilateral pleural effusion (*n* = 5). PET/CT and nuclear scintigraphy (111In) showed mildly to moderately avid uptake (e.g., SUV 3.9 in spleen, positive bilateral pleural effusions; *n* = 3), aiding differentiation from malignancy.

Histopathologic analysis across cases revealed the defining feature of extramedullary hematopoiesis as trilineage hematopoietic maturation, with erythroid precursors (glycophorin A/CD71+), myeloid elements (MPO+), and megakaryocytes (CD61/FVIII/CD42b+), often embedded within a reactive stroma exhibiting sclerosis, myxoid change, patchy fibrosis, hemosiderin deposition, and neoangiogenesis with elastica disruption. Atypical megakaryocytes, displaying nuclear pleomorphism, multinucleation, clustering, and dysmorphic contours, mirrored bone marrow findings and were ubiquitous, underscoring the clonal dissemination from myelofibrosis. Site-specific adaptations included arterial intimal occlusion in pulmonary EMH, adipocyte splaying in cutaneous proliferations, TMA-like renal sclerosis with intracapillary infiltrates, and serosal effusions harboring megakaryocytes amid reactive mesothelium; admixed lymphocytes, eosinophils, plasma cells, and histiocytes occasionally evoked inflammatory or neoplastic mimics, reliably excluded by targeted immunohistochemistry (negative cytokeratins, S100, SOX10).

A summary of patients’ characteristics is reported in Table 1.

**Table 1:** Characteristics of patients extrapolated from published case reports

	*N* (%) – [range] 47 patients
Median age, years	68 [43–82]
Sex, male: female	26:21
MF subtype:	
- Primary - post-PV - post-ET - NS	- 30 (64) - 7 (15) - 6 (13) - 4 (8)
Driver mutations:	
- JAK2 - CALR - MPL - Triple-negative - NS	- 19 (40) - 2 (4) - 1 (2) - 6 (12.7) - 19 (40)
Median time MF to EMH, years *(available for 25)*	4 (1–15)
Concomitant MF/EMH diagnosis	10 (21)
Prognostic risk (IPSS-MYSEC-PM)	
- Low - Int-1 - Int-2 - High - NS	- 1 (2) - 12 (26) - 11 (23) - 5 (11) - 18 (38)
Therapy at EMH	
- HU - JAKi - Other - None - NS	- 18 (38) - 6 (13) - 1 (2) - 8 (17) - 14 (30)
EMH sites:	
- Abdominopelvic - Thoracic - Cutaneous - Serosal - Endocrine - CNS - Other	- 13 (28) - 12 (26) - 6 (13) - 8 (17) - 5 (11) - 2 (4) - 5 (11)

### Treatment approaches for EMH

Management of EMH (available for 21 cases) was highly heterogeneous, but most patients received MF-directed systemic therapy with or without local treatments. Ruxolitinib-based regimens were the most frequently used approach, administered in 19 patients, either as monotherapy (*n* = 9) or in combination with other agents such as prednisone/EPO, hydroxyurea, radiotherapy, or in the context of long-term MF control. Among these, 6 patients were already receiving ruxolitinib at the time of EMH diagnosis, representing cases of breakthrough EMH despite ongoing JAK inhibition, a finding consistent with clonal escape or sanctuary biology. In the remaining cases, ruxolitinib was newly initiated or switched from non-JAKi agents (predominantly hydroxyurea) in temporal association with EMH diagnosis, suggesting therapeutic escalation prompted by disease progression. Busulfan plus prednisone was reported in 1 patient, hydroxyurea in 3, and fedratinib in 1, while other systemic strategies included a JAK2 plus navitoclax clinical trial and combinations with hypomethylating agents and venetoclax in a patient with concurrent blast-phase disease. Radiotherapy was used in 5 patients (10.6%), typically delivered as fractionated RT (e.g., 15 fractions of 200 cGy) or as localized RT to symptomatic lesions, and was sometimes combined with ongoing ruxolitinib. Splenectomy with adjunct hydroxyurea was reported in 1 case, and allogeneic HSCT was documented in 1 patient as definitive therapy, achieving complete remission of MF. In 16 cases (34%), treatment information was incomplete or not reported.

Response to treatment was variably described, but generally favorable when JAK inhibitors or local RT were employed. Among ruxolitinib-treated patients, several reports documented rapid improvement of EMH-related manifestations: resolution of dyspnea within one month, disappearance of pulmonary ground-glass opacities after approximately eight months, regression of ascites within three weeks, and normalization of liver and spleen size after six months of therapy. In some cases, symptomatic and radiologic control was maintained for years of continuous ruxolitinib. Ruxolitinib combined with RT led to a marked improvement in focal EMH after approximately 14 months, whereas splenectomy plus hydroxyurea induced fading of cutaneous or serosal lesions within days to weeks. Busulfan-prednisone resulted in only minimal improvement, and cases treated mainly with supportive measures or short-course cytoreductives often showed stable disease rather than full resolution. Across the cohort, explicit response descriptors clustered into partial responses (“symptomatically better”, “improved”, radiologic shrinkage), complete responses (reported after HSCT and in a subset of ruxolitinib/RT-treated cases), stable disease, and rare progression in the setting of blast transformation, illustrating that EMH is frequently controllable but not always eradicable with current therapies.

### Survival and prognostic factors

**Fig. 3:**
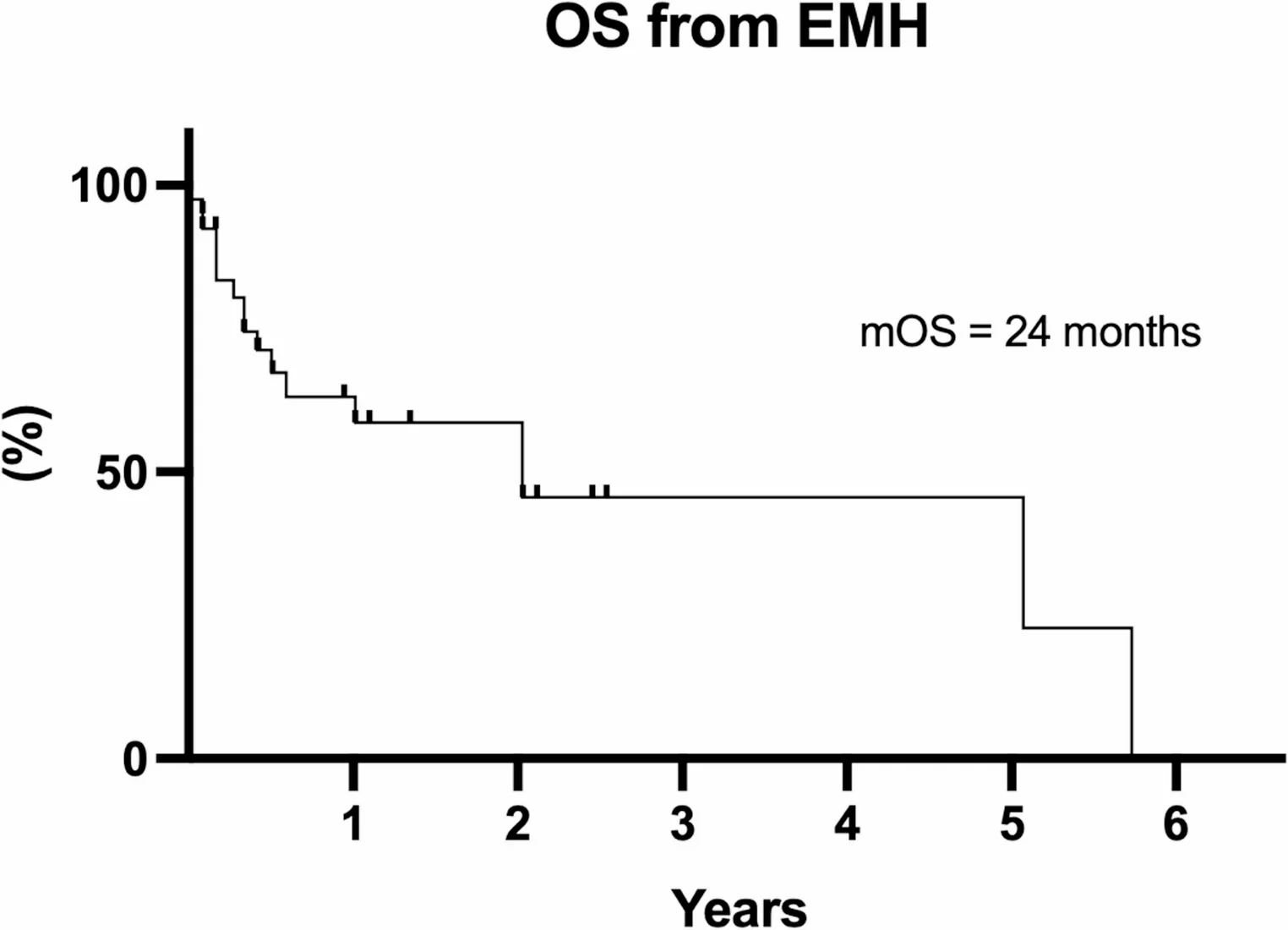
Overall survival (OS) for 40 patients with available follow-up (exploratory analysis only; publication date imputed as last follow-up where not explicitly reported). EMH: extramedullary hematopoiesis

Among the patients with available last follow-up, 18 deaths (38.3%) were recorded, with a median follow-up duration from EMH diagnosis to last follow-up of 5 months (ranging 1–69 months). Causes of death were directly attributable to disease progression or EMH complications in several instances, including myocardial infarction, cardiac tamponade, pleural effusion with respiratory failure, cardiotoxicity from presumed EMH cardiac infiltration, sepsis, blast transformation to AML, fungal pneumonia post-gastrectomy, portal hypertension, and acute heart failure. The remaining 22 patients (46.8%) were alive at the last follow-up. Survival data were incomplete or unavailable in 7 cases (14.9%). OS is represented in Fig. 3.

Exploratory subgroup patterns suggested worse prognosis for cases with rapid demise (< 1 month follow-up; *n* = 6, 40% of deaths), often tied to acute EMH-related organ failure (e.g., tamponade, respiratory collapse), whereas patients with longer observation (> 24 months; *n* = 5) included both survivors and those succumbing to late complications like sepsis or AML transformation. Data incompleteness precluded formal associations with EMH site, treatment type, or molecular features, but the high proportion of early mortality underscores EMH as a marker of advanced, multisystem disease burden in MF.

## Discussion

This systematic review of 47 cases delineates EMH in MF as a surrogate marker of advanced clonal dissemination and therapeutic refractoriness. The anatomic breadth - thoracic (25.5%), abdominopelvic (27.6%), serosal (17%), and cutaneous (13%) - mirrors the pathophysiologic mobilization of clonal progenitors via CXCL12/CXCR4 dysregulation, yielding ectopic trilineage foci with megakaryocytic atypia [50, 51]. Symptomatic presentation in 74.5% of cases (driven by dyspnea, abdominal pain, and constitutional decline) underscores EMH’s disruptive potential beyond splenomegaly, often mimicking infection, malignancy, or thromboembolism [52].

Ruxolitinib elicited responses in the majority of treated patients; however, 4 of 19 ruxolitinib-treated cases developed EMH as breakthrough disease under ongoing JAK inhibition, confirming sanctuary dynamics or clonal escape, particularly in the context of high-risk co-mutations such as SRSF2 and KRAS [53, 54]. Radiotherapy provided effective locoregional control as an add-on strategy, and HSCT achieved complete remission in one case, consistent with its role as the only potentially curative option [7, 8]. Overall mortality of 38.3% at a median follow-up of 5 months, with deaths attributable to EMH-related organ failure or AML transformation, positions atypical EMH as a DIPSS-independent marker of advanced disease burden.

Limitations include publication bias favoring dramatic presentations, incomplete treatment timelines (34%), missing survival data (15%), and a retrospective case-report design that precludes formal risk factor analysis. The survival data must be interpreted with caution. The follow-up duration was reported heterogeneously across publications, and publication date was imputed as the last follow-up in a subset of cases, introducing administrative censoring bias; this does not constitute a robust estimate of population-level prognosis. The inability to distinguish JAKi continuation from JAKi escalation in response to EMH limits therapeutic inference [3, 13, 54]. The high rate of missing driver mutation data represents a relevant limitation, precluding any molecular correlate analysis with EMH site, treatment response, or survival, reflecting publication-era conventions and multidisciplinary authorship. Future registries with omics/PET serials will test CXCR4 antagonists or antifibrotics (pelabresib), potentially recasting EMH from a fatal endpoint to a modifiable trait [55].

Based on the available evidence from this systematic review, the following pragmatic recommendations can be proposed:

- Imaging: Cross-sectional imaging (CT/MRI) should be performed in patients with persistent pulmonary or abdominal symptoms unexplained by splenomegaly; given the frequent multi-site involvement (documented in 3 cases), additional compartments should be systematically investigated.
- Biopsy: Histologic confirmation is recommended whenever feasible, particularly at ambiguous sites, to exclude malignancy and confirm trilineage hematopoiesis (MPO/CD61/glycophorin A).
- Therapy escalation: The development of EMH should be interpreted as a sign of disease escape from current cytoreduction, warranting a change in systemic therapy. Transition to (or substitution of) a JAK inhibitor is the preferred approach; if the patient is already on ruxolitinib, switch to an alternative JAKi (fedratinib, pacritinib, momelotinib) should be considered based on clinical phenotype.
- Prognostic awareness: The identification of EMH, particularly at atypical sites, is associated with poor overall survival (38.3% mortality, median follow-up 5 months) and accelerated risk of AML transformation. Patients should be informed; the presence of circulating blasts should prompt bone marrow reassessment and, when indicated, repeat NGS to identify high-risk co-mutations.
- Transplant evaluation: HSCT referral should be expedited, as EMH represents a marker of advanced disease; locoregional RT may serve as a bridge to transplant in selected cases.

## Conclusion

Atypical EMH in myelofibrosis signals advanced clonal escape with dismal prognosis. Multimodality imaging, biopsy confirmation, prompt escalation of JAK inhibitor therapy, and early transplant referral constitute the pillars of management. Prospective registries with mandatory molecular annotation are urgently needed to refine risk stratification and evaluate novel therapeutic strategies, including CXCR4-targeted agents and antifibrotics.

## Supplementary Information

Below is the link to the electronic supplementary material.

Supplementary Material 1

## Data Availability

The datasets generated during and/or analyzed during the current study are available from the corresponding author upon reasonable request.
