# Supplementary material for: A systematic review of 47 published cases of extramedullary hematopoiesis in myelofibrosis: clinical patterns, treatment responses, and prognostic implications
Source: Ann Hematol. 2026 Jun 30;105(10):423. doi: 10.1007/s00277-026-07146-2 (PMC13585788; doi:10.1007/s00277-026-07146-2)
Supplement: Supplementary file 1 [file 277_2026_7146_MOESM1_ESM.docx]

**SUPPLEMENTARY**

*Supplementary Figure 1. PRISMA 2020* flow diagram
